# Global Diversity and Phylogeny of the Asteroidea (Echinodermata)

**DOI:** 10.1371/journal.pone.0035644

**Published:** 2012-04-27

**Authors:** Christopher L. Mah, Daniel B. Blake

**Affiliations:** 1 Department of Invertebrate Zoology, National Museum of Natural History, Smithsonian Institution, Washington, District of Columbia, United States of America; 2 Department of Biological Sciences, Louisiana State University, Baton Rouge, Louisiana, United States of Ameica; 3 Department of Geology, University of Illinois at Urbana-Champaign, Urbana, Illinois, United States of America; J. Craig Venter Institute, United States of America

## Abstract

Members of the Asteroidea (phylum Echinodermata), popularly known as starfish or sea stars, are ecologically important and diverse members of marine ecosystems in all of the world's oceans. We present a comprehensive overview of diversity and phylogeny as they have figured into the evolution of the Asteroidea from Paleozoic to the living fauna. Living post-Paleozoic asteroids, the Neoasteroidea, are morphologically separate from those in the Paleozoic. Early Paleozoic asteroid faunas were diverse and displayed morphology that foreshadowed later living taxa. Preservation presents significant difficulties, but fossil occurrence and current accounts suggests a diverse Paleozoic fauna, which underwent extinction around the Permian-Triassic interval was followed by re-diversification of at least one surviving lineage. Ongoing phylogenetic classification debates include the status of the Paxillosida and the Concentricycloidea. Fossil and molecular evidence has been and continues to be part of the ongoing evolution of asteroid phylogenetic research. The modern lineages of asteroids include the Valvatacea, the Forcipulatacea, the Spinlosida, and the Velatida. We present an overview of diversity in these taxa, as well as brief notes on broader significance, ecology, and functional morphology of each. Although much asteroid taxonomy is stable, many new taxa remain to be discovered with many new species currently awaiting description. The Goniasteridae is currently one of the most diverse families within the Asteroidea. New data from molecular phylogenetics and the advent of global biodiversity databases, such as the World Asteroidea Database (http://www.marinespecies.org/Asteroidea/) present important new springboards for understanding the global biodiversity and evolution of asteroids.

## Introduction

### Introduction to Basic Biology and Morphology

The class Asteroidea (also known as starfish or sea stars) is one of the most diverse groups within the phylum Echinodermata, including nearly 1900 extant species grouped into 36 families, and approximately 370 extant genera. Asteroids occur at all depths from the intertidal to the abyssal (to approximately 6000 m) and are present throughout all of the world's oceans, but they are most diverse in the tropical Atlantic and Indo-Pacific regions [1], [2], [3]

All living asteroids have been regarded as members of the post-Paleozoic Asteroidea [4], [5], which have a Triassic (early Mesozoic) fossil first occurrence [6]. The taxonomy uses the term “Neoasteroidea” recognizing the modern Asteroidea (i.e., the post-Paleozoic Asteroidea) [5], [6]. Certain late Paleozoic asteroids show similar and intermediate morphology with the crown group, and these similarities have been treated differently [4], [5], [6].

Asteroids are dorsoventraly flattened with five to 50 rays projecting from a central disk. Each arm possesses a series of paired J-shaped ambulacral ossicles that occur along each arm radius. Tube feet emerge from pores present between ambulacral ossicles into a large ventrally facing open groove. These grooves all converge on the mouth, present on the bottom-facing side of the disk. Although supported as members of the asteroid lineage, concentricycloids (represented by the monotypic *Xyloplax*) show a highly divergent morphology that has suggested separation of *Xyloplax* from the other Asteroidea. This includes unpaired, non-overlapping ambulacral ossicles, tube feet in a single row, and adambulacral plates forming a peripheral disk series [7], [8]. As outlined below, this divergent morphology has led to a highly contentious discussion over the classification of *Xyloplax* within the Echinodermata.

In spite of the common names “sea star” and “starfish,” asteroids possess highly varied body shapes, including those that are sphaerical (e.g., *Podosphaeraster*), those that are pentagonal (e.g., *Sphaeriodiscus*) and others that are strongly stellate with very long arms and a nearly non-existent disk (e.g., *Zoroaster*). Body shapes range from highly inflated and cushion shaped (e.g., *Culcita*) to extremely dorso-ventral flattened with paper-thin bodies (e.g., *Anseropoda*). In many asteroids, a thick, fleshy (e.g., *Porania*) to gelatinous (e.g., *Hymenaster*) covering/layer has obscured the skeleton. Adult animal size varies from the tiny stichasterid *Allostichaster palmula* [9] with a disk to arm radius of about two to ten mm to immense members of the Asteriidae, such as *Evasterias echinosoma* and *Pisaster brevispinus*, which have both been recorded with armtip to armtip diameter of nearly 90 cm.

Other aspects of asteroid biology are diverse and are only briefly touched upon herein. Generalized overviews of asteroid biology can be found in [10], [11], [12], [13]. Jangoux [14] and Sloan [15] reviewed feeding biology and nutrition. Chia [16] and Koss and Rowe et al. [17] reviewed microscopic anatomy in asteroids and concentricycloids, respectively. Lawrence [18] reviewed eponymous structures in echinoderms, including several present in asteroids. Flammang [19], [20], Flammang et al. [21], [22] and Santos et al. [23] have provided several significant new contributions to our understanding of tube foot adhesion physiology. Valentincic [24] reviewed asteroid behavioral and responses to external stimuli. Chia and Walker [25] reviewed reproduction in asteroids. McEdward and Miner [26] reviewed larval and life cycle patterns.

### Importance

Asteroids occupy substantive ecological roles and are widely used subjects in developmental and experimental biology. Asteroids such as the North Pacific *Pisaster* have been important in ecological studies addressing the role of competition, reproduction [27], [28], [29], [30], [31] and community structure [32], [33], [34]. Paine [32] idealized *Pisaster* as the textbook example of a keystone species. *Pisaster ochraceus* has been seminal in revealing the importance of photoperiodic control of reproduction in marine animals [35], [36], [37], [38], [39]. Cold (e.g., *Asterias*, *Leptasterias*) and temperate-water (e.g., *Meyenaster*, *Coscinasterias*) asteriids continue to occupy prominent roles as model organisms in the fields of community structure [30] and feeding ecology [40]. *Asterias amurensis* is an introduced invasive [41], [42], [43], [44] and is perceived as a threat to Australia's shellfish industries.

Population outbreaks of the tropical corallivore *Acanthaster planci*, also known as the Crown-of-Thorns Starfish, led to widespread concern by coral reef conservation authorities as living reefs were devoured by massive numbers of *A. planci* [45], [46], [47]. Corresponding to their ecological importance, asteroids are also study subjects in marine pollution and toxicological studies. Uptake of toxic metals, PCBs, and the effects of oil have been tested on several genera, including *Asterias*, *Evasterias*, and *Coscinasterias* [48], [49], [50], [51]. Taxa in the Asterinidae have occupied a primary place of importance in developmental and reproductive studies [52], [53]. Additionally, sea stars have been used in a diversity of disciplines, including immunology [48], physiology [54], biochemistry [55], cryogenics [56], and parasitology [57]. Several asteroid species have become subjects in global warming and ocean acidification studies [58], [59], [60].

## Materials and Methods

Morphological terms and definitions follow Clark and Downey [2] and Blake [61]. Classifications begin with the morphological-based phylogenetic work of Blake [4]. Taxonomic diversity counts and conventions for species were obtained from the World Asteroidea Database [62] and from the Asteroid Names List [63], [64], [65], [66]. The classification used for this paper is present on Table 1. Images and data from the U.S Antarctic Research Program were also included [67].

**Table 1:** Breakdown of living taxa among the Neoasteroidea from Foltz and Mah [69], [181].

Superorder	Order	Family	# genera	# species
**Forcipulatacea**	**Forcipulatida**	**Asteriidae**	**35**	**178**
		Heliasteridae	2	9
		Stichasteridae	9	28
		“Pedicellasteridae”	7	32
		Zoroasteridae	7	36
		Total Forcipulatida	*60*	*283*
	**Brisingida**	Brisingidae	10	63
		Freyellidae	7	47
		TOTAL Brisingida	*17*	*110*
		TOTAL Forcipulatacea	77	393
	**Spinulosida**	Echinasteridae	8	133
		TOTAL Spinulosida	8	133
**Valvatacea**		**Poraniidae**	7	22
	**Valvatida**	Acanthasteridae	1	2
		Archasteridae	1	3
		**“Asterinidae”**	**25**	**147**
		Asterodiscididae	4	20
		Asteropseidae	5	6
		Chaetasteridae	1	4
		Ganeriidae	9	21
		**Goniasteridae**	**65**	**256**
		Leilasteridae	2	4
		Mithrodiidae	2	7
		Odontasteridae	6	28
		**Ophidiasteridae**	**27**	**106**
		**Oreasteridae**	**20**	**74**
		Podospherasteridae	1	6
		Solasteridae	9	51
		Caymanostellidae	2	6
		TOTAL Valvatida	187	763
**Valvatacea**	**Paxillosida**	**Astropectinidae**	**26**	**243**
		Benthopectinidae	8	69
		Ctenodiscidae	1	5
		Goniopectinidae	3	10
		Luidiidae	1	49
		Porcellanasteridae	12	30
		Radiasteridae	1	5
		Pseudarchasteridae	4	29
		TOTAL Paxillosida	56	439
		TOTAL Valvatacea	243	1224
	**Velatida**	Korethrasteridae	3	7
		Myxasteridae	3	9
		**Pterasteridae**	**8**	**116**
	**Concentricycloidea**	Xyloplacidae	1	3
		**TOTAL Species**	***343***	***1890***

“Quotation marks” indicate groups that were not supported as monophyletic.

**Boldface** indicates groups with large numbers of taxa.

We utilize “lineage” throughout the text as a general term to indicate a species or taxon and its nominal ancestor (and/or sister taxa where applicable) as opposed to the more context-driven term “clade”, which implies a distinct suite of synapomorphies for a branch taken from a specific phylogenetic hypothesis that may or may not exist for a specific clade.

## Results

### Taxonomic Diversity and Diversity Trends

In terms of total number of species, the Asteroidea (n = 1890 species) (Table 1) and the Ophiuroidea (n = 2064 species) [68] comprise the two most diverse classes within the living Echinodermata. Species counts and names utilized are those nominally accepted by the World Asteroidea Database as valid (or “accepted” by the database). Following Blake's [4] classification with modification by Mah and Foltz [69] the Valvatacea (Valvatida+Paxillosida) includes the greatest number of species (n = 1224), followed by the Forcipulatacea (n = 393 species), the Velatida (n = 145 species) and finally the Spinulosida (Echinasteridae), which includes 135 species (Table 1) [70]. Mah and Foltz [69] changed the composition of the Valvatacea to include the Solasteridae, but even with this difference (n = 51 species), from Blake [4], prior versions of the Valvatida included more genera and species than the Paxillosida [71], [64].

Species diversity is disproportionately distributed among the 36 families of living Asteroidea (Table 1). Seven families, Ophidiasteridae, Pterasteridae, Echinasteridae, Asterinidae, Asteriidae, Goniasteridae and Astropectinidae, each include more than 100 species. The Goniasteridae (n = 256) and the Astropectinidae (n = 243) include the largest number of species within the Asteroidea.

Species are not evenly distributed among genera. Within the Astropectinidae, *Astropecten* alone includes 43% (104/243) of the total number of species in the family [72]. The Goniasteridae includes 65 genera, most of which include multiple species [73]. At least eight goniasterid genera include more than 10 species. Several genera possess disproportionately high numbers of species relative to other genera within the family. *Henricia* includes some 68% (91/133) of the total known species in the Echinasteridae [70]. *Pteraster* (n = 45) and *Hymenaster* (n = 50) together account for 82% of the total number of species (n = 116) in the Pterasteridae [74]. The aforementioned illustrate the extreme cases, but several more examples of disproportionately high numbers of species/family exist. In nearly every instance of a genus with a disproportionately high numbers of species, these taxa include a global or widely distributed range. *Astropecten* is limited largely to tropical and temperate settings, but *Henricia, Pteraster*, and *Hymenaster* all have cosmopolitan distributions in cold to temperate water settings.

### Undescribed Biodiversity

It is of course difficult to evaluate how many living species remain to be discovered, but one estimate can be based on the rate of reognition in the relatively well-known and widely studied Goniasteridae, which contains the largest number of nominal genera and species in the Asteroidea (Table 1). Out of the total number of nominal genera (n = 65) and species (n = 256) in the Goniasteridae, approximately 12% (n = 31) of species and 14% (n = 9) of genera were discovered in the 21^st^ Century (2001 to present). Based on identified but undescribed museum goniasterid material (C. Mah, unpublished data), this would raise the total number of newly discovered genera to 37% and the number of species to 32%. This does not reflect a comprehensive survey of all museum collections but does suggest that a substantial number of asteroid taxa remain undescribed.

Another potential source of undiscovered/undescribed biodiversity is to be found in cryptic species. Several asteroid taxa, outlined in the “Diversity Trends”sections below, have now been identified as containing cryptic species, which are discrete lineages that are distinguished primarily based on molecular data that were not immediately recognizable from gross morphology. Widespread species are not uncommon among asteroids and it seems likely that this will further result in the identification of additional species diversity.

### Diversity Trends

Table 2 broadly categorizes asteroid families as occurring in “cold,” “temperate,” or “tropical” settings. These zones are broadly based on sea-surface temperatures, as outlined in Duxbury et al [75], with “cold” temperatures ranging between 0 and 5°C, “temperate” ranging between 5 and 15°C, and ‘tropical’ at 15° and higher. Deep-sea settings (below 200 m) are treated herein as part of “cold” temperatures. Assignment of taxa to these categories is based on occurrence data from the World Asteroidea Database [62] and other sources [63], [64], [65], [66]. However, given the wide-ranging distributions of taxa, some of these categories are continuous and/or display overlap.

**Table 2:** Cold-Temperate-Tropical Water Asteroid Occurrence.

**Cold Settings Only**	**Benthopectinidae**, **Brisingidae**, **Caymanostellidae**, **Ctenodiscidae**, **Freyellidae**, *Ganeriidae, **Goniopectinidae**, **Korethrasteridae**, **Leilasteridae**, **Myxasteridae**, *Odontasteridae, **Pedicellasteridae**, **Podosphaerasteridae**, *Poraniidae, **Porcellanasteridae**, *Pseudarchasteridae, **Radiasteridae,** **Xyloplacidae**, **Zoroasteridae**
**Primarily Cold w/minority shallow Tropical and/or Temperate Members**	*Astropectinidae, *Goniasteridae, *Pterasteridae, *Solasteridae
**Temperate & Cold-Water Occurrence**	*Chaetasteridae, *Stichasteridae
**Temperate, Cold & Tropical Occurrence**	*Asteriidae, *Asterinidae, *Echinasteridae, Heliasteridae, Luidiidae,
**Tropical Shallow Water Settings Only**	Acanthasteridae, Archasteridae, Mithrodiidae
**Primarily Tropical w/minority Cold-Water Members**	Asteropseidae, Asterodiscididae, *Ophidiasteridae, Oreasteridae

**Bold** indicates groups exclusively found in deep-sea settings (>200 m).

* indicates those with deep-sea members.

Out of the 36 families of living Asteroidea, 23 of those occur either exclusively or primarily in cold-water settings, six families occurred in temperate environments and seven were present primarily or exclusively in tropical water habitats. Taxa defined as “exclusively” cold-water were those families that occurred entirely in cold-water settings, such as the deep-sea or at high-latitudes. Those identified as “primarily” cold water have families that include 85% of taxa present in cold-water.

### Tropical Diversity Trends

Those families that are primarily or exclusively tropical, including the Acanthasteridae, the Archasteridae, the Asteropseidae, the Asterodiscididae, the Mithrodiidae, the Oreasteridae, and the Ophidiasteridae, are all members of the Valvatida, as observed by Blake [71] and Mah and Foltz [69]. The Ophidiasteridae and the Oreasteridae are the most taxonomically diverse asteroid groups throughout the tropical shallow-water Atlantic, and Indo-Pacific [2], [3]. Blake [71] argued that valvatidans, which prey on colonial or encrusting food items, are most diverse in the tropics as a result of defensive structures, such as armor and spines that protect against predators. Blake [1], [71] also posited that predatory asteroids, such as the Asteriidae that feed on active or non-colonial prey have morphological features associated with predation (e.g., wide tube foot grooves) that make them more vulnerable to predation in the tropics.

In a phylogenetic analysis of the Valvatacea, Mah and Foltz [69] found that some valvatidan clades, such as the Oreasteridae plus the Asteropseidae and Acanthasteridae, show diversification into the tropics relative to a temperate or cold-water water sister taxon (*Petricia*). Other sister taxon relationships (e.g., *Fromia* and *Lithosoma*) are similar.

Other asteroid genera, such as *Linckia*, *Nardoa*, *Ophidiaster*, *Tamaria* (Ophidiasteridae) and *Mithrodia* (Mithrodiidae) form “tropicopolitan” species complexes that occur in the tropical-shallow water Atlantic and Indo-Pacific [2], [3]. Preliminary data also suggest that genera such as *Echinaster* are widely distributed species complexes [76]. Taxonomic and geographic distribution data including, but not limited to, *Archaster* (Archasteridae), *Asteropsis* (Asteropseidae), *Fromia* (Goniasteridae), *Nardoa* (Ophidiasteridae), and *Pentaceraster* (Oreasteridae), suggest that they form widespread species networks across the Indo-Pacific/East Pacific region.

Some phylogeographic analyses of populations within a single tropical species have been performed. *Linckia laevigata* shows distinction between Indian and Pacific Ocean populations [77], [78], [79], [80]. Distinct lineages have been recognized in populations of the Indo-Pacific Crown-of-Thorns Starfish, *Acanthaster planci*, [81], [82] suggesting that multiple cryptic species are present throughout itswidespread distribution. Zulliger and Lessios [83] sampled 40 of the 150 speices in the widespread tropical genus *Astropecten* and discovered species complexes and likely cryptic species.

### Temperate Diversity Trends

Temperate water asteroids make up a minority of the total number of asteroids (Table 2) but nearly all families possess some representation, but even these genera mostly overlap with occurrence in either cold or tropical settings. For example, Waters and Roy [84] presented a global phylogeography of the temperate-water (but also tropical), fissiparous asteriid *Coscinasterias*. Waters' work also suggests the possibility of cryptic speciation in *Coscinasterias muricata* [85] and the ongoing divergence of populations (leading to species) in *Patiriella regularis* [86]. The asteriid *Leptasterias* occurs in temperate waters but has overlapping occurrence in cold-water setting. Full treatment of the *Leptasterias* species complex is below under the “Cold-Water Diversity Trends” section.

Brooding seems to be present in several temperate water taxa and has been included in several molecular phylogeographic studies. Naughton and O'Hara [87] presented a molecular phylogeographic analysis of the goniasterid *Tosia*. Their results identified a new species, *T. neossia*, which was independently supported by differences in reproductive behavior and larval mode. External morphological differences between *T. neossia* and *T. australis* were described, but had been overlooked in prior studies of the wider-ranging and variable species *T. australis*.

### Cold-Water Diversity Trends

A majority of asteroid taxa occur in cold-water and cold-temperate settings (Table 2), which include deep-sea and high-latitude habitats. Nineteen families occur exclusively in cold-water settings, and most of those are found exclusively in the deep-sea. Four families include genera that occur mostly in the deep-sea although some species occur in more temperate to tropical regions (e.g, *Astropecten* in the Astropectinidae or *Euretaster* in the Pterasteridae). Several asteroid groups with high numbers of species also range across different habitats. For example, the Goniasteridae, which shows the highest number of genera (n = 65) and the second highest number of species (n = 256), occurs widely in cold water (e.g., *Ceramaster*, *Evoplosoma*), temperate (e.g., *Tosia*) and tropical habitats (e.g., *Fromia*, *Neoferdina*).

Many abyssal asteroid taxa are widely distributed, and several genera show a global distribution [88]. *Porcellanaster* and other members of the Porcellanasteridae, for example, occur at abyssal depths in the Atlantic, Pacific, Indian, and Southern Oceans [2], [89]. Other taxa, such as *Freyella* and *Freyastera* spp. (Freyellidae, Brisingida) also occur at abyssal depths in the Atlantic, Pacific, Indian, and Southern Oceans [66], [90].

Some evidence suggests that at least some modern asteroid taxa have occurred in the past in shallower environments. Blake and Zinsmeister [91] described Eocene *Zoroaster* aff. *fulgens* fossils from shallow-water littoral sediments of Seymour Island, Antarctica. Zoroasterids are absent from the modern Antarctic asteroid fauna but *Zoroaster* spp. occurs in the Atlantic, Pacific, and Indian oceans to depths of nearly 5000 m [92]. Villier et al. [93] describes Cretaceous pterasterid ossicles from shallow-water sediments. Most modern pterasterids occur today in deep-sea settings. Although several members of living deep-sea asteroid groups are present in the fossil record [93], [94] from shallow-water sediments, there are few records of living asteroid groups with fossil occurrence in deep-sea sediments. Villier et al. [95] describes velatidans and forcipulataceans from deep-water sediments of the Jurassic Lagerstätte of La Volute-Sur-Rhône. The Japanese Miocene Morozaki formation is a Lagerstätte contains several well-preserved asteroid fossils [96].

Many widely distributed cold-water asteroid taxa show relatively conservative morphology and display relatively few discrete differences between species. Historical distinctions have often been based on continuous characters [2], [89], [90], [97]. However, studies addressing genetic divergence in the widespread Atlantic deep-sea species *Zoroaster fulgens* using COI and 16S regions of the mitochondrial genome [98] have found at least three different bathymetrically separated morphotypes that are reproductively isolated. Based on these results, it seems likely that determinations of deep-sea and especially abyssal asteroid diversity are likely underestimated.

Continuing taxonomic studies suggest widespread occurrence of several cold-water taxa, which were originally described as species occuring only in localized regions. For example, certain species of *Hippasteria*, including *H. trojana* and *H. hyadesi* were described as distinct species occurring in New Zealand and the Patagonian sub-Antarctic, respectively. Newer taxonomic accounts now regard these as widely occuring members of *Hippasteria phrygiana* [2], [99], [100]. Other cold water taxa that have widespread distributions and which show a pattern similar to *Hippasteria* include *Solaster* and *Lophaster* (both in the Solasteridae), *Henricia* (Echinasteridae), and *Pteraster* (Pterasteridae). This is in no way a complete list but merely touches on the most species-rich genera that would benefit from further study. These taxa suggest at least the possibility of cryptic species and the need to re-evaluate past synonymies with molecular phylogenetic methods.

Asteroids at high-latitudes in both the Arctic and the Antarctic include taxa that form diverse species complexes that show morphological intergradation along the taxon's range. For example, in the Arctic and adjacent Atlantic and Pacific regions, the asteriid *Leptasterias* includes approximately 38 nominal species [101], [102], [103], which show phylogeographic evidence of relatively recent trans-Arctic diversification and interchange [104], [105], [106]. The asteriid *Asterias* also shows this pattern [107].

Although asteroid diversity in the Antarctic is higher [108], there is less phylogeographic data available for species complexes present in the Southern Ocean. Janosik and Halanych [109] and Janosik et al. [110] have recently outlined new species and reconstructed phylogeographic relationships for the abundant and commonly encountered *Odontaster*, which occurs throughout the Antarctic region.

## Discussion

### Fossil History

Recent views on the most likely Paleozoic source for post-Paleozoic asteroids differ significantly [4], [5], [6], [111], but authors agree that the Paleozoic-Mesozoic transition marked a time of major extinction and re-diversification, thereby allowing separation in this paper based on time. Although the paper focuses on the Asteroidea, it is necessary to touch briefly on the origins and diversification of all early stellate echinoderms.

### Subdivisions of Paleozoic stellate echinoderms

**Figure 1:**
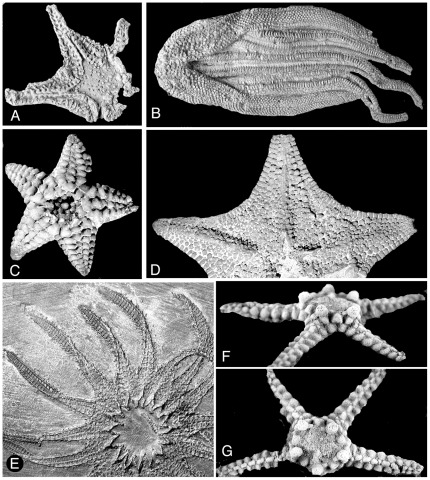
Paleozoic stem-group somasteroid and asteroids. A. *Ophioxenikos langenheimi* (Somasteroidea) Blake & Guensburg, X-4751. B. *Urasterella grandis* (Meek) USNM 40885. Ordovician. C. *Hudsonaster incomptus* (Meek) USNM 40882 Ordovician. D. *Jugiasspeciosus* (Miller and Dyer). MCS 10806. Ordovician. E. *Helianthaster rhenanus* Roember . PWL 1983-21, Devonian. F and G. *Paleaster clarki* Clarke and Swartz USNM 144825. Devonian.

All three recognized groups of radiate echinoderms or “Asterozoa,” the surviving Asteroidea (Fig. 1B–G) and Ophiuroidea and the extinct Somasteroidea (Fig. 1A) [112], first appeared in the fossil record during a comparatively brief interval of the Early Ordovician. Similarities among certain early members have led most paleontologists to think of asterozoans as monophyletic but based on differences among living representatives, some authors have favored disparate ancestries. This discussion treats only data from the fossil record and no attempt is made to resolve differences.

When named, the Somasteroidea was proposed as ancestral to both asteroids and ophiuroids. Since then, somasteroids have been seen as taxonomically cohesive [113] but their phylogenetic position has been both challenged [114], [115] and reaffirmed [115], [116]. Somasteroids can be separated from the surviving groups primarily on the basis of presence of a series of simple rod-like ossicles, so-called “virgals,” radiating laterally from each ambulacral ossicle. The first virgal is simple in all but one known somasteroid whereas it (or its equivalent) is differentiated as an “adambulacral” in asteroids and as a “lateral” in ophiuroids. The ambulacral column of asteroids is vaulted to form a permanent furrow and that of ophiuroids is vaulted only near the mouth frame. Based on ossicular configuration, the ambulacral column of somasteroids lies in the ventral plane, although it might have been capable of temporary vaulting to form a furrow [117]. Skeletal configurations appear to allow phylogenetic transformation from somasteroids to asteroids and ophiuroids, but conclusive evidence of sequencing is elusive.

### The Importance of Preservation in Understanding Asteroid Phylogeny

For a number of reasons, asterozoans are rare as fossils as compared with e.g., mollusks and brachiopods. Aspects of preservation and preservation and fossil preparation have been treated in many papers, including those of Jagt [94], Lehmann [118], Spencer [112], LeClair [119], and Villier [120] although general discussions are uncommon. Schuchert [121], Ubaghs [122], and Spencer and Wright [113] described constraints on asterozoan fossilization, and for Paleozoic representatives, Schuchert [121] included total then-known occurrences for each geologic period as well as number of species of various genera recorded from different modern nations.

Here, reasons why asteroids are poor candidates for preservation are discussed first, followed by consideration of whether or not the limited record might reflect limited diversity through geologic time. A skeleton of discrete, unfused elements, largely exposed life modes, and the limited paeloenvironmental range sampled in the rock record combine to work against asteroid preservation.

Asteroids today occur at all water depths and on indurated as well as particulate substrates; the fossil record is biased toward shelf habitats with particulate substrates, hence today many asteroids occur in settings only sparsely sampled in the geologic record. Asteroids are mostly epifaunal organisms and even for those living in favored habitats, preservation requires unconsolidated sediments for burial. Fossils can be found beneath storm deposits or within and beneath submarine sediment flows. Earthquakes trigger many sediment flows but downslope movement can be gravity-induced even on relatively low slopes.

The asteroid skeleton consists of a large number of proportionately small, unfused ossicles; this construction allows flexibility of movement. The dermal-skeletal layer of many asteroids can be tough enough to provide some resistance to dissociation, but once breached, decay rapidly proceeds and ossicles are dispersed. Soft organs in the proportionately large asteroid coelom doubtless attract scavengers, leading to typically relatively rapid destruction even among buried individuals. Intact asteroid preservation demands prompt burial without later disturbance. Most skeletally intact specimens are more or less collapsed, the comparatively tough body wall apparently prevented infiltration of sediment after internal organ decay.

Dense accessory arrays typical of asteroids present their own problems of interpretation. Accessories obscure the arrangement of the taxonomically important foundation ossicles but these smaller elements are also of taxonomic significance, and data are lost where they have been lost. Expressions of delicate pedicellariae are important in the taxonomy of many extant asteroids, but few are known from the Paleozoic, perhaps only because of loss during preservation.

Both small accessories and body wall ossicles obscure interior arrangement of the ambulacral column and especially of the mouth frame. As a result, internal appearance of the mouth frame is known for few fossil species. Specimen collapse under the weight of overlying sediment displaces skeletal elements and obscures relationships.

Preservation reflects selectivity for the more skeletally robust. For the better-known post-Paleozoic crown group fauna, the Astropectinidae and Goniasteridae dominate the fossil record, and many of the better-known Paleozoic representatives are also comparatively robust. Different authors have suggested predation pressure and burrowing intensity have changed through geologic time, and an increase in burrowing activity would be detrimental to preservation of the relatively delicate asterozoans.

Major geologic settings also bias samples. Certain of the more important Paleozoic European asterozoan faunas (e.g. Montaign Noire of France) accumulated in fine-grained, clastic sedimentary settings whereas many of the more important North American occurrences (e.g., Cincinnatian of Eastern United States) sampled carbonate-rich settings. Such depositional differences have preservational as well as paleoecological implications.

The many preservational constraints indicate that it is reasonable to interpret the fossil record of all asterozoans as a deeply biased sampling of what once existed. However, a second argument, the taxonomic diversity of known fossils, is available. The extant fauna provides a measuring tool for crown group (i.e. post-Paleozoic) occurrences. Although this record is dominated by the skeletally robust, known fossils record most of the more important living families, reflecting enduring diversity.

Paleozoic faunas, all belonging to stem groups, cannot be directly compared to a modern equivalent. Useful to their interpretation is the Early Devonian Hunsrück Slate fauna of Germany [118]. The Hunsrück Slate accumulated under geologically unique conditions [123], [124]. The asterozoan fauna includes both large and delicate species, many unknown elsewhere. Although a single occurrence, the Hunsrück diversity range is (at least subjectively) parallel to if not greater than that of the modern fauna.

Fossil preservation differs significantly among specimens, and important features are not available in all specimens. A sampling of the diversity of Paleozoic somasteroids and asteroids is illustrated in Figure 1.

### Origins of the Asteroids

Ancestry of the asteroids has been sought in two groups of early echinoderms, the extinct Edrioasteroidea and the Crinoidea (however, early crinoids were quite different from surviving representatives). The edrioasteroid hypothesis has been generally preferred; Smith and Jell [125] provided a recent perspective, and Zhao et al. [126] published reconstructions of certain edrioasteroids that might be suggestive of an asteroid ancestor. The crinoid hypothesis of Fell [127] received some early support but it was soon challenged [128] based on morphologic discontinuities, although recent discoveries appear to narrow differences [129], [130]. Mooi [131] reviews several different echinoderm phylogenetic hypotheses.

Like asteroids, edrioasteroids and crinoids have skeletons constructed of a large number of small, radially aligned plates or ossicles, and these similarities offer fertile ground for phylogenetic speculation. However, no known fossil bridges a morphological gap that begins with a skeleton of closely abutted elements and progresses to a flexible asteroid descendent. Further, the life-habit transition from a sessile or attached edrioasteroid or crinoid living with its mouth directed into the water column to a free-living descendent living with the mouth directed to the substrate is not bridged. Asteroid ancestry might lie within either edrioasteroids or crinoids, but much remains to be learned. Although the work of Fell [117], [127] was then not yet available, G. Ubaghs, one of the most important students of early echinoderms during the 20th century, found asterozoans to be of uncertain derivation [122], and his assessment remains sound.

Efforts at locating an asteroid ancestor of necessity focus on available fossils, but the comparatively very few yet very significant discoveries of early echinoderms of Guensburg and Sprinkle [129], [130], which were based on more than twenty years of intensive field research, clearly testify to the importance of what remains unknown. Further, both the biased fossil sampling of crown group asteroids as well as the echinoderm composition of the Early Devonian fauna of the unique Hunsrück Slate of Germany [123], [124], including many taxa unknown from other localities, attest to incomplete overall sampling. Reconstruction of the origins and early diversification of stellate echinoderms must be based on very limited and biased evidence with much early history likely to remain forever unknown.

### Paleozoic Asterozoa: Important Classification Schemes

The meager fossil record has led to comparatively few taxonomic arrangements of Paleozoic asterozoans. For ordinal-level taxa, Spencer [112] provides the starting point. In this paper and following his own monographic work [132], Spencer purposed the extinct Somasteroidea as the ancestor of the surviving ophiuroids and asteroids.

Ubaghs [122] used terminology and concepts taken from Spencer [112], including the Somasteroidea. H. B. Fell [117], [127], [133], [134] proposed *Platasterias*, as a surviving somasteroid genus, although this interpretation is no longer generally accepted [135], [136]. Fell also posited a crinoid ancestry for living asterozoans, and he argued that extant asteroids can be used to help infer an ancient transition between crinoids and asteroids. Spencer and Wright [113] used the subordinal Paleozoic terminology of Spencer [112] as well as some new terms, and they accepted the phylogenetic ideas of Fell. In a survey treatment emphasizing German fossils, Müller [137] endorsed the three-fold subdivision of Spencer as well as the incorporation of Paleozoic fossils into extant orders. R. V. Kesling [138], [139], [140], [141], [142], [143], [144], [145] revisited the interpretations of Spencer and Wright [113]; these authors treated family through subclass rankings as well as a number of genera, some of them new. They also evaluated certain of the difficulties in the recognition of ossicular homologies. In a brief study, McKnight [146] treated the full history of asteroids and somasteroids based on collections of extant taxa and the literature for fossils; ophiuroids were not included. This author focused on projecting characters of living asteroids onto groupings of Paleozoic fossils, including soft-tissues and ontogenetic data, as well as certain skeletal expressions. He subdivided asteroids into two new superorders, both ranging from the Paleozoic that show the strong influence of the ideas of Fell [117] and of Spencer and Wright [113]. Shackleton [114] provided a phylogenetic analysis and classification of all asterozoans, but limited her treatment to Ordovician representatives. This author did not use subdivisions between the class and familial levels for either asteroids or ophiuroids.

The coverage of Ubaghs [122] was comprehensive for Paleozoic genera whereas his treatment of post-Paleozoic taxa was less complete. Spencer and Wright [113] provided a comprehensive listing of known fossil and extant genera. The compilation of Schuchert [147] provides valuable data for any survey of Paleozoic genera.

### The Paleozoic Asteroidea: Complexities of Classification

Palaeontologists have traditionally regarded the Asterozoa as monophyletic but treatment within the group has varied significantly. Schuchert [121] recognized asteroids and ophiuroids as subclasses of Stelleroidea, and both used the asteroid terminology of Sladen [148], “Phanerozonia” (enlarged marginal ossicles) and “Cryptozonia” (reduced marginal ossicles). Schuchert [121] stressed his usage as descriptive subdivisions rather than as evolutionary markers. Schuchert [121] concluded that designation of taxa between the subclass and familial levels was premature. Schöndorf [149] recognized a class Auluroidea, on par with asteroids and ophiuroids. Kesling [139] embraced the auluroid concept whereas other workers have assigned these genera to the Ophiuroidea.

In their publications, W.K. Spencer, G. Ubaghs, H.B. Fell, R.V. Kesling, and D.G. McKnight all wove their arrangements of Paleozoic asteroids into the existing ordinal-level classification of the crown group. Spencer and Wright [113] included a historical summary of major papers leading to their arrangement.

Cited stratigraphic ranges and phylogenetic diagrams of Spencer and Wright [113] and especially of Ubaghs [122] indicate skepticism on the part of these authors over ranges extended from Paleozoic into the Mesozoic. Ubaghs [122] recognized only one such family, the Arthrasteridae. He assigned Carboniferous *Calliasterella* and *Protarthraster* to the Arthrasteridae, along with Cretaceous *Arthraster*, but he then dotted his range chart, seemingly questioning the arrangement. Ubaghs [122] treatment of predominantly crown-group asteroids was brief, but he did include Devonian *Jaekelaster* Sturtz and Mississippian *Compsaster* Worthen and Miller in the modern order Forcipulatida; he did not suggest familial assignments for these genera and his range chart does not clearly reflect his text suggestion.

Spencer and Wright [113] were somewhat more assertive in their arrangement. These authors recognized 12 suborders, five of which were thought to span the Paleozoic-Mesozoic boundary. They extended ranges of three small families (Palasterinidae, Calliasterellidae, Compsasteridae) across this boundary; however none of the three likely represents a monophyletic cluster [4], [6], [150]. The other two suborders of Spencer and Wright [113] were represented by families found on one side of the Paleozoic-Mesozoic boundary or the other, but not spanning it.

Shackleton [114] did not use taxon levels between the class and familial levels and no ranges crossing the Paleozoic-Mesozoic boundary were recognized. Although differing on stemward events in the crown group, Separate authors [1], [4], [5], [6], [111] have agreed that no extant ordinal-level taxon should be extended downward into the Paleozoic. Basic asteroid configuration and behavior have endured since early in class history, allowing much evolutionary convergence through geologic time.

Beginning with Paleozoic representatives, Blake and Hagdorn [6] proposed the subclass Ambuloasteroidea based primarily on presence of podial pores between successive ambulacral ossicles and offset placement of ambulacrals and adambulacrals, the former gradually emerging in different Paleozoic lineages, the latter extremely rare; the Neoasteroidea was treated as an infraclass within the Ambuloasteroidea. The Ambuloasteroidea provides an objective starting point in the search for the progenitors of the crown group.

### Life Modes of Paleozoic Asteroidea

Rigorous data on ancient life modes are few. Paleozoic asteroids have been collected exclusively from marine rocks, including both quiet and more active depositional settings, and from both soft and firm substrates. All ancient asteroids appear to have been bottom-dwelling organisms. Certain living asteroids bury themselves at shallow depths beneath the surface, and Spencer [112] suggested that somasteroids were burrowing organisms; however, no asteroid exhibits a bilateral shape typical of active burrowing organisms such as irregular echinoids. Many living asteroids have been observed partially or fully covered with sediment and it seems plausible that Paleozoic asteroids behaved in a similar fashion.

Modern asteroids include suspension-feeders, detrital feeders, and predators on varied prey. Blake and Guensburg [151] reported the Paleozoic *Promoplaeaster* with its arms wrapped around a pelecypod in a manner similar to modern day asteriids, suggesting an early occurrence of this feeding behavior. Herringshaw et al. [152] provided useful summary of life habits of multiarmed species and the difficulties of their interpretation. Blake and Rozhnov [153] argued ancient asteroids likely were capable of broad ranges of behavior comparable to those found today.

### Classification and Phylogeny of Post-Paleozoic Asteroids

#### Classification

Relatively few of the early syntheses of asteroid classification integrated fossil and living members in a phylogentic context [113], [122], [154]. Clark and Downey [2] presented the latest historical review of asteroid classification, emphasizing Atlantic taxa.

The late 19th and early 20th centuries were the “classic” period of morphologically based monographic studies of the systematics of modern asteroids. Authors consistently separated the forcipulate groups as recognized here from the remainder, and the Paxillosida gradually emerged as well, although there has been some instability of assignment (e.g., *Radiaster*, *Pseudarchaster*). The remaining groups proved more controversial, and remain so. Most influential were the ordinal concepts of Perrier [155], whose work was embraced in the widely cited Treatise of Spencer and Wright [113].

Concepts of modern higher classification among the living Asteroidea began with Viguier [156] and Perrier [155], [157] with subsequent contributions by Sladen [148] and Fisher [158]. Viguier established early groupings based on the nature of the skeletal mouth frame. Perrier [155] heavily emphasized pedicellariae as diagnostic for his four groups, the Forcipulatae, Spinulosae, Valvatae, and Paxillosae. Sladen [148] developed a different classification that largely emphasized marginal plates and regrouped the higher classification into the Phanerozonia, which included several families displaying prominent marginal plate series versus those in the Cryptozonia, which included those families that displayed more inconspicuous marginal plate series. Fisher [158] modified Sladen's classification and established three orders, the Phanerozonia, the Spinulosa, and the Forcipulata, which were in turn each subdivided into several suborders (e.g, the Paxillosa, Valvata, Notomyota) which accommodated previous classification schemes established by Perrier [155] and others and came to be heavily used throughout the 20^th^ Century.

### Phylogeny Inferred from Morphology

One of the earliest and best-known discussions of asteroid phylogeny began as a heated exchange between Mortensen [159], [160] and MacBride [161], [162], [163]. Their debate focused on the identity of the ancestral asteroid taxon. Mortensen assigning the “ancestral condition” to the Astropectinidae in part based on the absence of both a brachiolaria stage and suckered tube feet and MacBride arguing essentially that these are derived features in both astropectinid and luidiids reflected their occurrence on shallow, unconsolidated bottoms. Other workers surveyed by Mortensen [159] found that not only were the Paxillosida thought of as the “primitive” group, but also the Asterinidae and the “Spinulosa.” MacBride's contentious position did not definitively provide an alternative taxon as the ancestral asteroid but demonstrated the difficulty of interpreting “ancestral” versus “derived” characters.

The Mortensen-MacBride debate laid the foundation for the subsequent hypotheses of Fell [117], [133], [134], which suggested that the luidiid *Platasterias* was a living member of the Paleozoic Somasteroidea. This supported interpretation of the Paxillosida as the “primitive” or ancestral asteroid taxon and was embraced by Spencer and Wright [113]. Subsequent work [4], rejected Fell's interpretation of *Platasterias* and the Luidiidae as ancestral, but the debate over the Paxillosida as the group displaying the most “primitive” characters continued into modern discussions of asteroid phylogeny.

Although the “Paxillosida is primitve” discussion remains one of the best-known phylogenetic debates, there are several examples of other, less prominent, pre-cladistic, evolutionary hypotheses within the Asteroidea. Döderlein [164], [165] provided phylogenetic hypotheses for various species groups within both *Astropecten* and *Luidia*. H.L. Clark [166] provided early ideas on relationships among the *Heliaster* species complex in the tropical East Pacific. Madsen [167] presented ideas and an evolutionary tree regarding the interrelationships of the deep-sea Porcellanasteridae.

The modern phylogenetic paradigm for the Asteroidea begins with the cladistic-based hypotheses of Blake [4] and Gale [5]. Although the phylogenetic hypotheses significantly differ from one another, both show a well-supported modern Asteroidea as a discrete post-Paleozoic clade. In some respects, the work of Blake and Gale mirror those of MacBride [161], [162], [163] and Mortensen [159], [160] in that Gale [5], [111] advocates a primitive Paxillosida (Mortensen's position) whereas Blake argued that these characters should be interpreted as derived (MacBride's position).

An important distinction between the two phylogenetic hypotheses is that whereas Gale presented the Paxillosida as primitive, Blake emphasized the ambiguity of identifying any extant asteroid group as basal is misleading [4] (p. 515). Paleozoic lineages of asterozoans and early asteroids suffered extinction during the Permian-Triassic transition interval. Twitchett and Oji [168] summarized that all living echinoderms (including asteroids) underwent an important evolutionary bottleneck during this interval with subsequent recovery and diversification within the Triassic. Fossils are few but offer important insight [6], [169]. Extinction is an important component of understanding the early history of crown-group asteroids. Thus, our knowledge of early lineages within the Neoasteroidea is very poorly understood and the determination of a “primitive” taxon, such as the Paxillosida, is misleading and is an oversimplification of a complex but obscure history for which multiple taxa were likely present [6], [169], [170] but not reconciled within the reconstruction of a phylogeny which has only surveyed available living and fossil taxa.

Blake [4] showed the Forcipulatacea as the sister taxon to the remainder of the surviving asteroids, a separation that has been historically observed in primary asteroid monographs [148], [155], [158]. However Blake [4], [6] has emphasized that even those tree topologies that incorporate available fossils depends on the sampling of a scanty fossil record. It is important to note that divergence might be such that the common ancestor of all surviving asteroids would no more be assignable to a surviving taxon grouping below the class level than is the early Paleozoic common ancestor.

### Phylogeny Inferred from Molecular Studies

Early molecular studies, such as that published by Wada et al. [171] and the combined analysis of Lafay et al. [172] are consistent with Gale's [5], [111] assertion that the Paxillosida were primitive. However both Wada et al. [171] and Lafay et al. [172] included relatively few taxa and used conveniently sampled, local species as avatars for large, highly diverse groups (such as the highly diverse Valvatacea). Many of their sampled species, including *Astropecten* and *Luidia*, have since been shown to occur on highly derived branches [69], [83]. Gale [111] has continued to argue Mortensen's perspective of a “primitive” Paxillosida in spite of phylogenetic evidence to the contrary from morphology [4], [61] and recent evidence from several molecular studies [69], [173], [174] that have shown the Paxillosida in derived positions.

Knott and Wray [175] presented one of the first, well-sampled phylogenetic analyses of the Asteroidea from COI, mtRNA and previously collected ribosomal gene sequences. Janies [176] presented a combined evidence tree of the Echinodermata, which supported the Asteroidea as monophyletic, but did not recover any consistently monophyletic groupings.

Matsubara et al., [177] determined the Solasteridae as the sister group to the Asterinidae and subsequently revisited the phylogenetic relationship of the Forcipulatida to other asteroids [174]. Waters et al., [178] addressed molecular relationships within the Asterinidae. Yasuda et al. [179] reported complete mitochondrial genome sequences for the Crown-of-thorns starfish *Acanthaster*, and provided a COI phylogeny showing *Acanthaster*+*Oreaster* in addition to other asterinids on a valvatidan clade as the sister group to two paxillosidans (*Astropecten* and *Luidia*) rooted against a forcipulate (*Pisaster*), an echinoid and a holothurian. Foltz et al. [180] supported the monophyly of the Forcipulatacea using combined mitochondrial and nuclear sequences.

Mah and Foltz [69] reconstructed a comprehensively sampled phylogeny of the Valvatacea which supported the sister group relationship between the Asterinidae and Solasteridae as determined by Matsubara et al. [177] as well as supporting stemward relationships for the Poraniidae and the Velatida (Pterasteridae, Myxasteridae, Korethrasteridae). Although basal relationships were not well supported, the Paxillosida was not supported among basal taxa within the Valvatacea relative to a Forcipulatacean outgroup (Fig. 1) [69]. A subsequent phylogenetic analysis of the Forcipulatacea [181] further supported forcipulate monophyly, re-established the Stichasteridae, and clarified relationships among groups within the Asteriidae and among the Forcipulatacea.

### Diversity among the Living Asteroidea

All living asteroids, termed Neoasteroidea by Gale [5], are phylogenetically distinct from those in the Paleozoic [5], [6]. Gale [5] named the Post-Paleozoic Asteroidea as the Neoasteroidea. Based on construction of the ambulacral column, Blake and Hagdorn [6] recognized the Neoasteroidea at the infraclass level within a subclass Ambuloasteroidea

**Figure 2:**
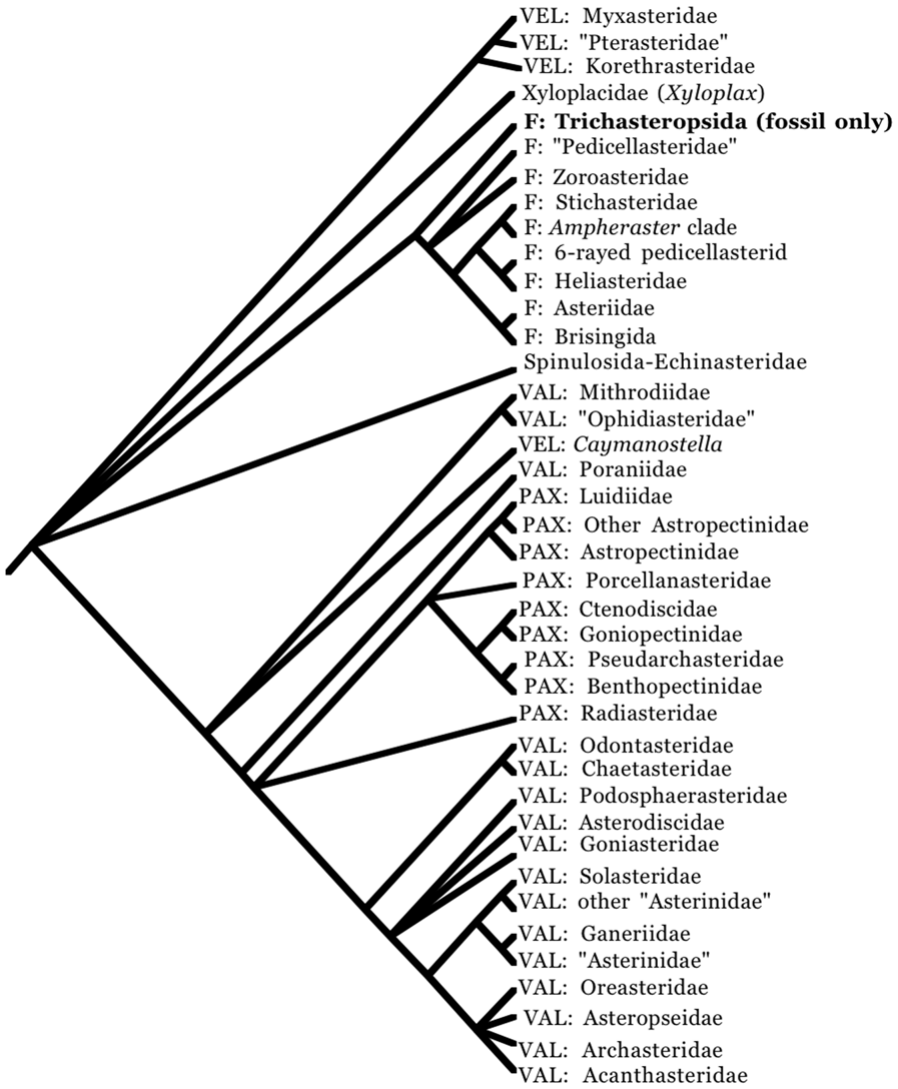
Summary diagram of phylogenetic tree. Topology from combined trees of Mah and Foltz [69], [181], Janies et al [173], and Blake [4]. “Asterinidae” refers to paraphyletic clades as outlined by Mah and Foltz [69].

Figure 2 summarizes phylogenetic perspectives from Foltz and Mah [69], [181], Blake [4], and Janies et al. [173]. Polytomies are present where phylogenetic data is incomplete or ambiguous but the diagram assumes a monophyletic Neoasteroidea. Groupings used below reflect discrete phylogenetic lineages rather than traditional taxonomic units. The Velatida has not found full support as a member of the Spinulosacea and, except for *Caymanostella*, is retained separately.

Mah and Foltz [69], [181] presented a 3-gene phylogeny that has further clarified relationships and classification in the Forcipulatacea and the Valvatacea. These include the paraphyly of the Asterinidae along with several proposed taxonomic changes, namely the assignment of the Solasteridae to the Valvatida and placement of some ophidiasterids in the Goniasteridae, the new position of the Poraniide, the paraphyly of the Pedicellasteridae and others, which are outlined in discussions below. Gale [111] has proposed the Forcipulatida as rooted among several valvatidan taxa as the “Tripedicellaria.” This is a classification with no precedent in the historical literature from morphology [4], [148], [154] and it has found no support with other recent molecular data [69], [173], [177], [179]; it therefore is not followed herein.

### The Forcipulatacea

The Forcipulatacea is a diverse, primarily cold-water (some temperate and tropical members are known) lineage of modern asteroids that occur in all of the world's oceans from the intertidal to the deepest abyssal depths (>6000 m). The Forcipulatacea includes 393 species in 77 genera (Table 1) [182], which ranks them as among the most diverse of the Asteroidea. Forcipulataceans are most diverse at high-latitudes with rich faunas in the Arctic and especially in the Antarctic.

**Figure 3:**
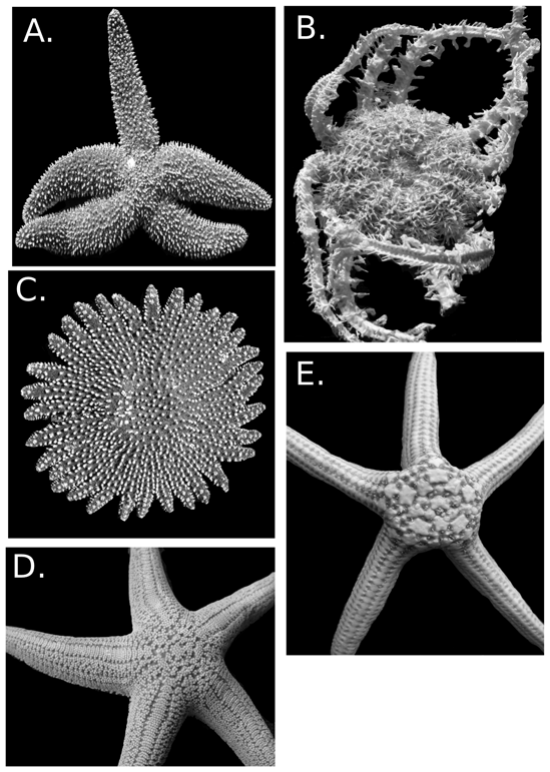
Forcipulatacean diversity. A. *Asterias forbesi* (Asteriidae) USNM 43197 B. *Odinella nutrix* (Brisingida) USNM E13561. C. *Heliaster cumingii* No number. D. *Stichaster striatus* (Stichasteridae) USNM 1085979. E. *Doraster constellatus* (Zoroasteridae) USNM E23145.

Although the Forcipulatacea display a wide range of morphologies (Fig. 3), taxonomists traditionally have found them to be readily separated from the remainder of the crown group. Characters helping to characterize forcipulataceans but not found in all members include the presence of distinct 3-part “forcipulate” pedicellariae (although pedicellariae vary among taxa), four rows of tube feet; foreshortened (or “compressed”) ambulacral and adambulacral ossicles, the latter alternating in furrow profile in taxa with four rows of tube feet; a reticulated dorsal skeleton; a well-developed adoral carina (abutted adambulacral plates adjacent to the mouth, the proximal skeleton recessed to form a so-called actinostome); small mouth-angle ossicles; the longest actinal series adjacent to the marginals rather than adjacent to the adambulacrals; and a small disk with thick, tapering arms.

Most historical accounts [113], [158] have set apart the Forcipulatacea or “forcipulate” asteroids (i.e., the Forcipulatida+Brisingida) from the other members of the Asteroidea. This is a position that has been further supported by modern phylogenetic treatments of morphology [1], [4], [173], [174] and is reflected in Fig. 2. Gale [111] has placed forcipulates in a derived position within taxa historically regarded as members of the Valvatida. This position has not found historical agreement and is not followed by the treatment herein.

Monophyly of the Forcipulatacea itself has been relatively uncontroversial with support from traditional taxonomy [101], [183], morphology-based phylogenetic studies [4], [5] and molecules [173], [174], [177], [180], [181]. Subgroupings within the Forcipulatacea have encountered more difficulty, especially those associated with the Asteriidae, such as the Labidiasteridae [101], [113], [183], [184]. Mah and Foltz [181] provided the taxonomic foundation for the summary below.

The Forcipulatacea, particularly the Asteriidae (e.g., Fig. 3A), includes some of the most heavily studied and most familiar of marine invertebrates in ecology and environmental biology. Relevant taxa include *Pisaster ochraceus*, which has become an iconic representative of the keystone species concept as outlined by Paine [32], [33], [34] and *Asterias amurensis*, which has been introduced to southern Australia as a pest species that threatens endemic shellfish [41],[42],[43],[44]. The Atlantic *Asterias rubens* and *Asterias forbesi* have been among the most familiar of ecological subjects in marine biology studies [185], [186]. As important ecological members, asteriids such as the European *Asterias rubens*, the North Pacific *Evasterias troscheli*, and the temperate South Pacific *Coscinasterias muricata* have also been used as subjects in several oil pollution studies [50], [51], [187 respectively].

### Diversity Within the Forcipulatacea

Mah and Foltz [181] supported six primary lineages within the Forcipulatacea. This includes the Asteriidae (e.g., Fig. 3A) [188] the Brisingida (e.g., Fig. 3B) [189], a modified Heliasteridae (Fig. 3C) [190], the Stichasteridae (Fig. 3D) [191], the Zoroasteridae (Fig. 3E) [192] and a paraphyletic “Pedicellasteridae” [193]. Many of the traditional asteriid subgroupings outlined by Fisher [101], [183], which were raised to family-level by Clark and Downey [2] and Clark and Mah [66], were not supported as monophyletic, although some of the Northern/Southern Hemisphere taxonomic divisions in his identification keys were observed. Some groups, such as the Labidiasteridae, are artificial and have been dismantled [181], [184]. Basal relationships among forcipulatacean lineages were not well supported, but higher-level groups were recovered from the analysis.

The Brisingida (Fig. 3B) [189] is a clade of exclusively deep-sea asteroids possessing a small disk with tightly articulated plates and six to 20 elongate arms, which are extended into the surrounding water column and used for feeding [194]. Brisingids are suspension feeders that utilize needle-like spines with dense coverings of pedicellariae to capture tiny crustaceans and other food particles [195], [196]. They are found between 100–6000 m and have been reported from all oceans, except the Arctic. The Brisingida have been repeatedly supported as monophyletic by morphology [4], [181], [184], [197] and DNA [180], [181], and include 110 species in 17 genera [189]. Within the Brisingida, the monophyletic Freyellidae (47 species in seven genera) [198] occupy a much deeper bathymetric range than non-freyellids [197]. The Brisingidae (63 species in ten genera) [199] itself is likely paraphyletic and includes *Brisingaster*, *Novodinia*, and *Odinella*, which are likely basal within the overall brisingid clade relative to *Brisinga* or other non-freyellids [200]. The Zoroasteridae (Fig. 3E, 7 genera, 36 species) [192] and the “Pedicellasteridae” (7 genera, 32 species) [193] both occur only in the deep-sea (bathyal to abyssal depths) and are phylogenetically basal among extant forcipulataceans. The basal location of these taxa was consistent with Blake [1] who supported Jurassic “asteriid” fossils as closely related to zoroasterids and pedicellasterids. Zoroasterids possess a single row of marginals, a character present in Paleozoic and early transitional asteroid fossils from the Triassic [4], [6]. Pedicellasterids display numerous plesiomorphic characters, such as biserial tube foot rows, an absent or reduced adoral carina and a weakly developed abactinal skeleton. Mah and Foltz [181] did not recover the Pedicellasteridae as a monophyletic group, instead finding support for multiple basal lineages within the Forcipulatacea, suggesting that the term “pedicellasterid” is best applied as a grade within forcipulates, rather than a monophyletic family. A phylogeny of the Zoroasteridae [92] separated the more imbricate zoroasterids, such as *Zoroaster* and *Cnemidaster*, which occur from bathyal to abyssal depths, from zoroasterids with reticulate skeletons, such as *Myxoderma*, which occur at shelf to bathyal depths. This suggested diversification of the more derived imbricate taxa, such as *Zoroaster*, into the deep-sea.

The Heliasteridae (Fig. 3C, nine species in two genera) [190] includes the tropical shallow-water *Heliaster*, which occurs throughout the Pacific coast of Mexico and South America and *Labidiaster*, which occurs in the South Atlantic and in the adjacent Southern Ocean. *Heliaster* comprises a species complex in the East Pacific region [166] with some ecological importance [32]. Pliocene fossils from Florida have indicated that this complex at one time occurred over a much larger region [201]. *Labidiaster annulatus* in the Southern Ocean is a benthopelagic predator [202], [203]. Mah and Foltz [181] recovered a sister-group relationship between *Heliaster* and *Labidiaster*, which provided the basis for synonymy of the artificial and paraphyletic Labidiasteridae within the Heliasteridae. Mah [184], Foltz et al. [180], and Mah and Foltz [181] dismantled the Labidiasteridae, showing that each of its members was assignable to phylogenetically distant lineages.

Two of the most ecologically important and diverse groups within the Forcipulatacea, are the Asteriidae (with most species in the Northern Hemisphere) (Fig. 3A, 35 genera, 178 species) [188] and the mostly Southern Hemisphere Stichasteridae (Fig. 3D, 9 genera, 28 species) [191]. In spite of being phylogenetically distant from one another, the Asteriidae and Stichasteridae include taxa that apparently occupy similar if not convergent ecological niches in intertidal and shallow-water marine ecoystems [32], [204], [205], [206], including “keystone” positions as predators of bivalves and other mollusks.

Multiple lineages are present within the Asteriidae and the Stichasteridae. Four major lineages are present in the Asteriidae including the genus *Sclerasterias* a Boreal clade, which contains Northern Hemisphere cold-temperate water taxa, such as the Pacific-Arctic-Atlantic *Leptasterias* species complex [104], [105], [106], and two sister clades, the Pan Tropical and Antarctic asteriids. The Pan Tropical asteriid clade is composed of taxa such as *Coscinasterias*, *Meyenaster*, and *Astrometis*, which occur at low-latitudes in tropical (non-reef) to temperate settings. Antarctic asteriids occur at high latitudes in the Southern Ocean and adjacent regions and are the most diverse of the Antarctic asteroid fauna. High-latitude asteriids include brooding taxa, such as *Diplasterias*, *Lysasterias*, and *Anasterias* [108].

The Stichasteridae occur on two major lineages. One primarily shallow-water cluster, including *Stichaster*, *Cosmasterias*, *Smilasterias*, and *Allostichaster* which occur in an austral distribution in South America, South Africa, and Australia/New Zealand and its sister lineage which is composed primarily of deep to cold-water taxa with widespread distributions, such as *Neomorphaster*.

### The Paxillosida and “Notomyotida”

**Figure 4:**
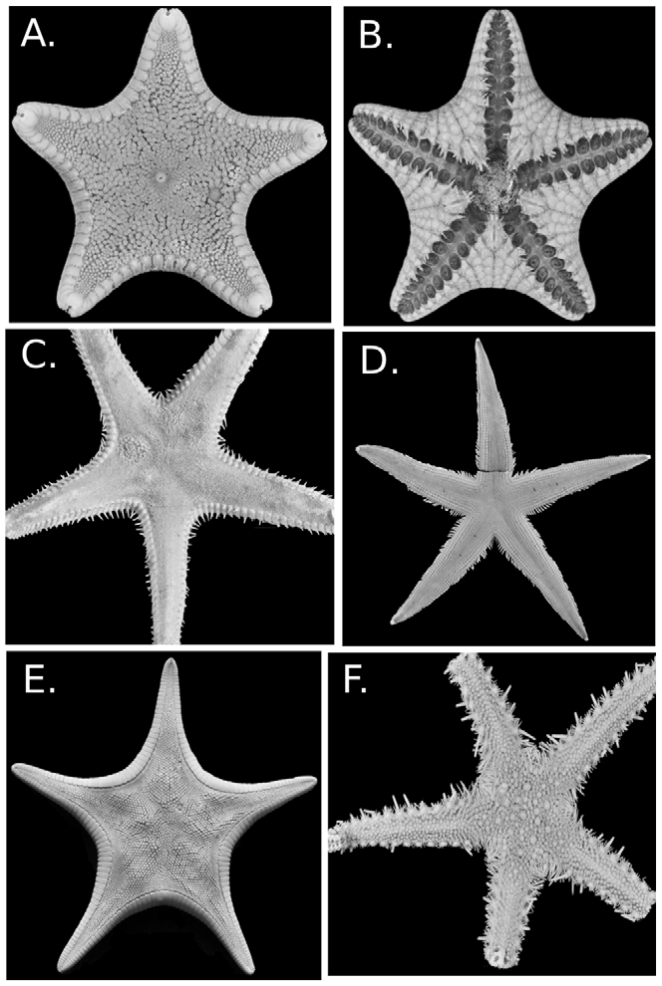
Paxillosida (including Benthopectinidae) diversity. A. *Ctenodiscus australis*, abactinal surface USNM 37148 B. Same specimen, showing actinal surface and fasciolar grooves. C. *Dytaster grandis* USNM E15959 D. *Luidia clathrata* USNM 8507 E. *Pseudarchaster parelii* USNM 1085998 F. *Luidiaster antarcticus* USNM 1121741.

The Paxillosida (Fig. 4), including the Benthopectinidae, occurs at depths ranging from littoral habitats (e.g., *Astropecten* in the Astropectinidae occurs at 0–2 m in some settings) to the deepest abyss (>5000 m) (e.g., the Porcellanasteridae). Most of the Paxillosida are primarily cold-water and are well represented in the deep-sea as well as at high latitudes (Arctic and Antarctic) but include diverse, shallow-water tropical to temperate water taxa as well (e.g., *Astropecten, Luidia*). The review herein follows the phylogeny of Mah and Foltz [69] and includes the Benthopectinidae and the Pseudarchasteridae as members of the Paxillosida.

The primary life mode of taxa within the Paxillosida, with the exception of the Benthopectinidae, involves burial or ploughing through unconsolidated sediment [61], [207]. Examples of characters that have been considered adaptations to life in sediment and simultaneously synapomorphies for many members of the Paxillosida include paxillate plates (abactinal, marginal and actinal), pointed tube feet, superambulacral plates, cribiform organs, the presence of an anal cone, and actinolateral fasciolar channels.

The Paxillosida includes both detritivores and predators of mollusks and other invertebrates [14], [15], and many spend part or most of their lives buried. Paxillosidan life modes are associated with poorly consolidated sediment bottoms. Some groups, such as the goniopectinids, ctenodiscids, and porcellanasterids are detritivores that live buried in or under mud [208] whereas others live buried under surface sediments but are predatory on mollusks and other invertebrates [14]. Ecology in most of the Paxillosida is poorly understood, but observations of *Astropecten*, *Ludia*, and other paxillosidans suggest complexity and ecological importance [209], [210], [211], [212], [213]. Although *Pseudarchaster* appears to be more phylogenetically distant from the other Paxillosida, it shows a generalized detritivore/predatory feeding life mode similar to astropectinids [14].

Little is known regarding the biology of the Benthopectinidae. Jangoux summarizes stomach contents from four taxa, which suggests they are either predators/sediment feeders/detritivores. Blake [214] and Clark and Downey [2] have speculated that benthopectinids used muscles to hold up their arms in the water column for suspension feeding and interpreted their well-developed arm spines as defensive adaptations to predators that have limited them to deep-water. Available images of benthopectinids do not suggest burrowing or show arms extended into the water column.

### Diversity Within the Paxillosida (and “Notomyotida”)

Mah and Foltz [69] used three genes (12S, 16S, and histone H3) to reconstruct the phylogeny of the Valvatacea, and recovered a Paxillosida that was composed of traditional members (e.g., Astropectinidae, Goniopectinidae, Luidiidae, etc.) but also several groups displaying intermediate morphology. This included the Benthopectinidae and the Pseudarchasteridae as sister taxa to a clade containing the Goniopectinidae and the Ctenodiscidae. The Luidiidae was recovered as the sister lineage to one containing multiple astropectinids, including *Macroptychaster*, *Lonchotaster*, *Leptychaster*, *Dipsacaster* and the radiasterid, *Mimastrella*. Although the Porcellanasteridae was not sampled in Mah and Foltz's [69] analysis, it was supported as one by Blake [4] and is considered as a member of the Paxillosida herein.

The Porcellanasteridae (12 genera, 30 species) [215], Goniopectinidae (3 genera, 10 species) [216], Ctenodiscidae (Fig. 4A,B, 1 genus, 5 species) [217] as well as most members of the Astropectinidae (Fig. 4C, 26 genera in 243 species) [72] all occur primarily in deep-sea settings (∼100–4000 m). Common to all of these families are genera that have a cosmopolitan (or nearly so) distribution. For example, the porcellanasterid, *Porcellanaster ceruleus* displays a cosmopolitan distribution [63], [89]. Multiple genera within the Astropectinidae possess widespread, deep-sea distributions at bathyal to abyssal depths, including *Dytaster*, *Leptychaster*, *Lonchotaster*, *Persephonaster*, *Plutonaster*, and *Psilaster*. *Ctenodiscus*, the sole member of the Ctenodiscidae is present throughout the world's ocean basins, occurring from the Arctic to the deep-sea tropics to the subAntarctic. Many of these taxa display few characters or characters that differ only gradually across their range.

In contrast to the deep-sea Paxillosida, there are two genera, *Astropecten* and *Luidia* (Fig. 4D) with large numbers of species that occur in temperate and tropical settings. Although both genera occur across a wide range, most taxa are primarily shallow-water and live in relatively coarse sediments compared to other deeper-water Paxillosida, which occur in finer, deep-sea muddy bottoms. Döderlein produced a taxonomic overview of both genera [164], [165]. Zulliger and Lessios [83] analyzed 117 specimens of *Astropecten* belonging to 40 species from around the world, using 12S, 16S and COI genes, and identified three main clades in the Indo-Pacific, the Neotropics, and the eastern Atlantic and Mediterranean, which displayed morphological convergence and several species complexes, such as the *A. polyacanthus* complex in the Indo-Pacific.

The Benthopectinidae (Fig. 4F) and the Pseudarchasteridae (Fig. 4E) were supported by Mah and Foltz [69] as sister taxa and both have shown close morphological resemblance/affinities to the Goniasteridae [4]. The Pseudarchasteridae (e.g., *Pseudarchaster*, *Paragonaster*) includes 29 species in four genera [218], whereas the Benthopectinidae (e.g., *Benthopecten*, *Nearchaster*) includes 69 species in eight genera [219]. Both families occur primarily in deep-sea (shelf to abyssal) or high-latitude/polar settings and include many widely distributed taxa.

### The Poraniidae (Sister clade to Valvatida+Paxillosida)

**Figure 5:**
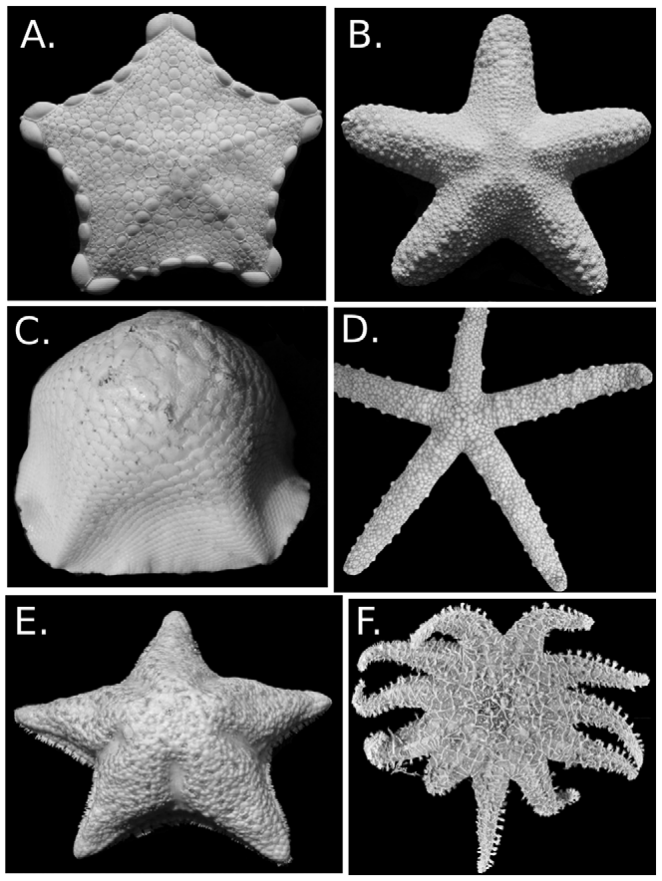
Diversity within the Valvatacea. A. *Pentagonaster pulchellus* (Goniasteridae) USNM E9756 B. *Pentaster obtusatus* (Oreasteridae) USNM C. *Tremaster mirabilis* (Asterinidae) USNM E46295 D. *Nardoa tuberculata* (Ophidiasteridae) E16509 E. *Porania pulvillus* (Poraniidae) USNM 11035 F. *Crossaster campbellicus* USNM 1122950.

Mah and Foltz's [69] work placed the Poraniidae (Fig. 5E), which had historically been a member of the Valvatida, as the sister clade to a Valvatida+Paxillosida dichotomy, thus removing it from the Valvatida [4]. This is consistent with the morphology-based phylogeny of Blake and Hagdorn [6] that showed a Poraniidae+*Noriaster* clade as sister to solasterids, asterinids, echinasterids, paxillosidans, and goniasterids.

The Poraniidae includes 22 species in seven genera [220], which are distributed in cold-water settings throughout the world, including high-latitude/polar regions and the deep-sea. Poraniids inhabit primarily cold-water settings, primarily at high latitudes or in the deep-sea [221] and are distinctive asteroids with a typically thickened fleshy body wall that has obscured the endoskeleton and made classification of the group difficult [222]. Our understanding of poraniid biology is largely based on information derived from two polar species, *Porania antarctica* and *Porania pulvillus* and the temperate water *Poraniopsis* spp.

Feeding in known poraniids [14] suggests that most are detritivores or predators. Bowden et al. [223] shows *Porania antarctica* feeding on stalked crinoids in the Antarctic. Ericsson and Hansson [224] observed *P. pulvillus* feed on octocorals, a brachiopod, and several ascidian species. Dearborn [202] observed *P. antarctica* feed on detritus, but sometimes preying on sea urchins. Gemmill [225] described ciliary suspension feeding in *P. pulvillus*, although further confirmation of this behavior has not been observed.

### The Valvatida

In terms of numbers of taxa at all levels, including families, genera, and species, the Valvatida (Fig. 5) is the most taxonomically numerous within the Asteroidea and as such, life modes and ecology are diverse. Mah and Foltz's [69] analysis found that the Solasteridae (Fig. 5F), which have historically been assigned to the Spinulosida [158] were nested within the clade containing the Asterinidae, which has further extended the limit of diversity within the Valvatida.

Life modes in the Solasteridae are different from other Valvatida. Jangoux [14] outlined feeding of multiple solasterid taxa, including *Solaster* and *Lophaster*. Most solasterids are primarily predators of other mobile or otherwise active invertebrate taxa, including gastropods, cnidarians, and other echinoderms, such as holothurians and asteroids [226], [227]. Blake [1] has interpreted the decalcified skeletons, and wider, more open tube foot grooves as associated with active predation, but also as a more vulnerable body form, which may limit solasterids from tropical regions

Non-solasterid valvatidans possess a generalized life mode, feeding primarily on sessile prey items. Jangoux [14] summarized various benthic prey including encrusting algae, organic biofilm, foraminiferans, sponges, bryozoans, hydroids, corals, gorgonians, multiple anthozoans, ascidians, and various detrital food sources (e.g., fecal pellets, dead fish, urchins, etc.).

Different valvatidan taxa are involved in complex ecological interactions, especially with cnidarians. *Acanthaster planci*, the Indo-Pacific Crown-of-Thorns Starfish is an important predator of scleractinian reef corals [47]. Goniasterids are important predators of shallow-water pennatulaceans [228] as well as deep-sea corals [100], [229].

Blake [1], [4], [71] argued that the success of tropical-shallow-water valvatidans, such as the Oreasteridae, Ophidiasteridae, Acanthasteridae and others was related to multiple characters, such as spines, narrow tube foot furrows, thick granulated epidermis, and well-developed body skeletons, that provided defenses against predators. Many of these tropical shallow-water taxa are abundant and are significant members of the ecological communities of these regions [230]. The growth and biology of several several tropical valvatidans (e.g., oreasterids, archasterids) has become of increasing concern [231], [232], [233], [234] as many of these species are taken for tourist and aquarium/pet industries [235]. *Linckia laevigata*, a brilliant blue ophidiasterid is among one of the most heavily trafficked species in pet and tourist trades [236], [237].

Several high-latitude valvatidans, such as those that occur in the Antarctic, including odontasterids, ganeriids, and solasterids, are predators on sessile prey, such as sponges but also on other echinoderms [202], [238]. Several Antarctic valvatidans, such as the odontasterid *Odontaster validus*, *Perknaster fuscus*, and *Acodontaster conspicuus* are ecologically important [239]. *Odontaster validus*, probably is the most intensively studied of Antarctic asteroids [39], [109], [110], [238], [239], [240].

The Asterinidae have served as model organisms in developmental and reproductive biology as well as in ecology and conservation studies. *Patiria miniata*, the Pacific Northwest bat star, common along the west coast of North America, has become one of the primary model organisms in developmental gene studies [241], [242]. Building on this research, other taxa of asterinids have been heavily used in a wide variety of studies, including life history evolution [243], gene expression [244], and the evolution of reproduction and larval development [245], [246]. Many asterinids occupy intertidal and nearshore habitats and are important subjects in the study of marine ecosystems [247], [248] especially in the context of their reproductive biology [249].

Based on observations of feeding in most shallow-water to temperate species, most asterinids appear to be detritivores or omnivores that feed on encrusting organisms, algae, decaying corpses, and other detritus [14]. At least one asterinid, the New Zealand *Stegnaster inflatus*, has developed elaborate ambush methods for capturing mobile prey [250].

### Diversity Within the Valvatida

The Valvatida is a diverse lineage that includes some of the most taxon-rich families within the Asteroidea. Most members of the Valvatida possess a well-defined marginal plate series that frequently outlines the periphery of the body. In addition, boundaries between plates are relatively well-defined and the disk is large with well-defined actinal regions, and a relatively heavily calcified or otherwise modified skeleton. Valvatidan taxa include the Acanthasteridae, Archasteridae, Asterodiscididae, Asteropseidae, Goniasteridae (Fig. 5A), Oreasteridae (Fig. 5B), Ophidiasteridae (Fig. 5D), and the Odontasteridae (Fig. 5D and see Table 1). Other taxa supported as valvatidans display substantial departure from this overall body plan, including the Asterinidae (Fig. 5C), Ganeriidae, and Solasteridae (Fig. 5F). No published molecular data is available for the enigmatic Podosphaerasteridae, but morphological studies [4], [251] have consistently placed it among the Valvatida.

Several members of the Valvatida are important members of tropical shallow-water settings, such as reefs, mangroves, and sandy bottoms [230]. Valvatidans typically found in these regions include *Culcita* (Oreasteridae), *Acanthaster* (Acanthasteridae), *Protoreaster* (Oreasteridae) and *Archaster* (Archasteridae). Many are widely distributed throughout the Indo-Pacific. For example, *Acanthaster planci* is present from the coast of Baja California, north to Hawaii and Japan, and is present west to the east coast of Africa in the Indian Ocean [47]. Although groups such as the Oreasteridae (Fig. 5B) and the Ophidiasteridae (Fig. 5D) are known primarily from tropical shallow-water habitats [3], [252], [253], many individual members of these groups occur in deeper water. Mah [254] and H.E.S. Clark [255] describe deep-water oreasterid taxa (*Astrosarkus* and *Acheronaster*, respectively). Deep-sea ophidiasterids, such as *Tamaria* are well documented [2], [99] but poorly understood.

Cold-water valvatidans are highly diverse (Table 2). The Goniasteridae (Fig. 5A) [73] includes the greatest number of genera (n = 65) and species (n = 256) within the living Asteroidea. Most goniasterids occur in cold-water settings, primarily the deep-sea (e.g., *Litonotaster*, *Nymphaster*), but also in Antarctic and subAntarctic settings (e.g., *Pergamaster*) [256] in cold to temperate water intertidal zones (e.g., *Tosia*). Some goniasterids (e.g., *Fromia*, *Anchitosia*) are also widely distributed in tropical habitats [229], [257], [258]. Although the Goniasteridae includes more taxa than almost any other family of asteroids, relatively few comprehensive reviews are available [99], [259], [260], [261].

The Odontasteridae [262] and Ganeriidae [263] occur mainly in the Antarctic and subAntarctic as well as in the deep-sea. Odontasterids were supported as basal to the clade containing all of the Valvatida and possess several characters, such as paxillate abactinal and marginal plates, that suggest shared, possibly plesiomorphic characters with the Paxillosida. Ganeriids are more derived and show close relationship to asterinids and solasterids.

Mah and Foltz [69] supported the Asterinidae as a member of the Valvatida and presented a potentially significant shift in asteroid classification by showing the traditional Asterinidae as a paraphyletic assemblage. This has changed the perception of the Asterinidae, from that of a traditionally derived, monophyletic grouping to a plesiomorphic grade relative to the more derived morphology in the Solasteridae and Ganeriidae. Some asterinids are shown as sister taxa to ganeriid and solasterid clades whereas others are present on more stemward positions on the Valatida clade.

The Asterinidae (Fig. 5E) [264] and Solasteridae (Fig. 5F) [265] are morphologically significantly different from the other Valvatida. The Solasteridae have historically been considered members of either the Spinulosida or the Velatida [4]. Many solasterids, including *Solaster* and *Crossaster*, possess anywhere from six to 15 arms and possess reticulated, lightly calcified skeletons compared to other valvatidans. Most solasterids occur in cold to temperate water settings, but one genus, *Seriaster*, occurs in the tropical shallow water settings of New Caledonia [266].

**Figure 6:**
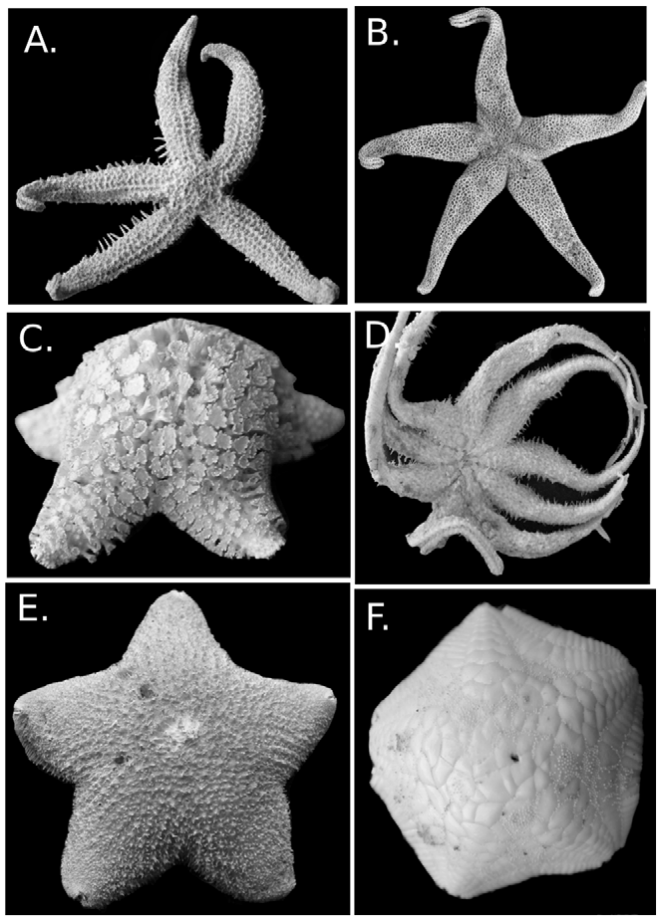
Forcipulatacea, Spinulosidan, Velatidan Diversity. A. *Ampheraster marianus* (“Pedicellasteridae”-Forcipulatacea) USNM E16024. B. *Henricia obesa* (Echinasteridae) USNM 1120449. C. *Remaster gourdoni* (Korethrasteridae) USNM E 47646. D. *Myxaster sol* (Myxasteridae) Yale Peabody Museum 36040 E. *Diplopteraster multipes* (Pterasteridae) USNM 5530. **F**. *Caymanostella spinimarginata* (Caymanostellidae) USNM E 27575.

The Asterinidae are highly diverse, occupying different habitats and displaying a diverse, but consistent, series of body forms. Asterinids are morphologically distinctive with flattened bodies that range from swollen and thickened (e.g., *Patiriella*) to nearly parchment-like in thickness (e.g., *Anseropoda*) with body forms that range from pentagonal (e.g., *Meridiastra*, *Tremaster*) to more stellate (e.g., *Nepanthia*) and can have five to nine arms. Abactinal plates are either flat, scalar, and overlapping or are more crescentic-in shape approaching an appearance of chain-mail armor. O'Loughlin and Waters [267] summarized a full range of asterinid body forms. Most asterinid diversity is known from shallow tropical to temperate-water settings (e.g., *Aquilonastra*, *Asterina*, *Parvulastra*) with relatively small adult size (diameter = 0.5 to 2.0 cm). Temperate to cold-water forms, such as *Patiria*, *Patiriella*, and *Stegnaster* are larger in size (from eight to 15 cm in diameter). Cold-water asterinids, such as *Tremaster* (Fig. 6C) and *Anseropoda* show the largest sizes among the Asterinidae and are widely distributed in deep-sea settings, showing nearly global distributions with occurrence in Antarctica, the Indian Ocean, the central Pacific, Hawaii, and in the North Atlantic [267].

The Podosphaerasteridae includes the sole genus *Podosphaeraster*, which has been recorded from the deep-sea in the Atlantic and Pacific Oceans. *Podosphaeraster* is unique among asteroids in having a highly divergent, round, sphaere-like body shape, resulting in ongoing interest regarding plate homologies [251],[268]. A.M. Clark [269] originally assigned *Podosphaeraster* to the Mesozoic Sphaerasteridae. Blake [4], [270] disagreed with this assignment. Fujita and Rowe [251] later re-classified *Podosphaeraster* in a new monotypic family, the Podosphaerasteridae. Both Blake [4], [270] and Fujita and Rowe [251] outlined close affinities between *Podosphaeraster* and the Goniasteridae, but this relationship has not been fully tested with molecular data and although its classification is stable, many phylogenetic questions remain.

The Caymanostellidae (Fig. 6F) are dorsoventrally flattened with scalar plates and are known primarily from deep-sea wood substrates [271], [272] and superficially appear similar to concentricycloids. Little to nothing is known regarding caymanostellid biology or ecology. Given the unusual morphology of caymanostellids, especially given their resemblance to concentricycloids, determination of the phylogenetic position and classification of caymanostellids has been an active field of study.

Morphological evidence from fossils and modern forms have argued for an affinity with *Tremaster* and related tremasterines within the Asterinidae [272], [273], which suggested placement within the Valvatida. Caymanostellids were absent from Gale [5], [111] but were supported among the Velatida by Blake [4]. *Caymanostella* is supported among the Valvatida as the sister taxon to *Archaster* in the molecular tree of Janies et al [173]. Morphological and molecular evidence appears to support the Caymanostellidae as members of the Valvatida by several of the published studies. However, given uncertainties regarding taxon sampling, this relationship is expressed in Figure 2 as part of a valvatacean polytomy and among the Valvatacea in Table 1.

### The Spinulosida

Phylogenetic efforts have changed the taxonomic composition of the Spinulosida in the 20^th^ Century from the more inclusive definition outlined in Fisher [158] to the more restricted monotypic Spinulosida, which included only the Echinasteridae [4]. The Echinasteridae contains a large number of species (n = 133) assigned to a relatively small number of genera (n = 8) (Table 1) [70]. The largest genera are the tropical, shallow water *Echinaster*, which includes 27 species [274] distributed in the Atlantic, Indian, and Pacific Oceans and the globally distributed cold-temperate water *Henricia*, which includes 91 species [275]. *Henricia* is found at high-latitudes and in deep-sea settings.

Echinasterids generally possess a small disk with narrow, elongate arms and body wall plates that are similar in appearance, forming a reticulated mesh. Variably sized spinelets are found on every plate, these vary in shape from conical and thorny to fine and more nearly cylindrical.

Feeding in echinasterids varies, but a survey of known species of *Henricia* and *Echinaster* suggest that they consume microalgae, biofilms, and encrusting invertebrates, such as sponges and tunicates. Anderson [276] provided an important account of feeding and the digestive system in *Henricia*.

### Diversity Within the Spinulosida

*Henricia* (Fig. 6B) includes 68% (91/133) of the total number of echinasterid species [275], a total strikingly disproportionate as compared to totals for other genera assigned to the family. *Henricia* is present in cold-water settings, such as in the deep-sea (to >1000 m) and in polar or subpolar regions [2], [65], [158], [275]. Many species of *Henricia* intergrade morphologically such that clearly defined boundaries are difficult to recognize [2], [158], [277]. Molecular and reproductive approaches to the systematics of *Henrica* have led to the discovery of new cryptic species, such as *Henricia pumila* from the well-studied intertidal regions of the Pacific Northwest [278].

*Echinaster* displays an issue similar to the one observed in *Henricia*. It is a wide-ranging species that shows intergradation and problematic species boundaries. Other echinasterid genera, such as *Metrodira*, *Plectaster* and *Rhopiella*, include far fewer species that have more restricted range distributions.

### The Velatida

Based on the molecular phylogeny of Mah and Foltz [181], three families –the Pterasteridae (Fig. 6E) [74], Myxasteridae (Fig. 6D) [279], and Korethrasteridae (Fig. 6C) [280] are upheld as members of the Velatida, a classification that differs from Blake [4] who placed the Solasteridae and the Caymanostellidae within the Velatida.

The molecular phylogenies of Mah and Foltz [69], [181] support a monophyletic Velatida occupying a position separated from the Forcipulatacea and Valvatacea. Taxon sampling from within the Velatida was incomplete [181], but monophyly for the Korethrasteridae was supported. *Asthenactis*, a myxasterid was upheld within the Pterasteridae, but full taxon sampling remains ongoing. Morphology-supported phylogenies [4], [5], [111] have placed the Velatida in derived positions with the velatida embedded or closely related among taxa within other clades. The molecular phylogeny of Janies et al. [173] supported *Xyloplax* along with *Pteraster* and *Hymenaster* on a sister clade to the other living Asteroidea.

Pterasterids, korethrasterids, and myxasterids occur almost exclusively in cold-water settings, with most present in bathyal to abyssal or high-latitude habitats. The former three families possess paxillae covering the body surface. Oral plates are prominent, marginal plates weakly developed or absent, and pedicellariae are absent.

A unique, canopy-like secondary dorsal covering, a so-called “supradorsal membrane,” is found in the Pterasteridae. The supradorsal membrane is supported at the tips of highly elongate paxillae, and it encloses a so-called “nidamental cavity” between the membrane and the dorsal surface of the body. The nidamental cavity is open to the sea along the margins of the body and also through an opening or so-called osculum at the center of the dorsal disk. Muscles move water through the nidamental cavity, bringing fresh water to the respiratory papulae in the dorsal body wall. The supradorsal membrane is relatively sturdy, even canvas-like, in shallower-water *Pteraster* but more delicate and almost gelatinous in deeper-water *Hymenaster*. Pterasterids also have the ability to secrete copious amounts of apparently protective mucus [281], [282].

Reproductive biology in pterasterids is atypical and includes brooding [283], [284] and pelagic direct development [285], .

Food items of korethrasterids and myxasterids have yet to be recorded, but observations of *Pteraster* spp. show that they feed primarily on sponges [14], [227]. Gut contents of the deep-sea pterasterid *Hymenaster* suggest that they consume sediment and other detritus [14].

### Diversity Within the Velatida

Nearly all velatidans are found in deep-water and polar habitats. Many species assigned to individual genera are similar in overall appearance and are geographically widely distributed.

The Myxasteridae (example in Fig. 6D) is composed of 9 species in 3 genera [279] and possess five to ten arms, a weakly calcified skeleton, and occur at bathyal/abyssal depths (750–3800 m depths) in the Atlantic and Pacific oceans. They are rarely encountered animals with fewer then fifteen specimens known for the family in collections throughout the world. The Korethrasteridae (Fig. 6C) occurs in Arctic, Antarctic and deep-sea regions, and includes only 7 species assigned to 3 genera [280]. Although korethrasterids are not as rare as myxasterids, biology of the group, including feeding and reproduction remain poorly understood. Korethrasterids consistently possess five rays with paxillar plates covering the body surface

Taxonomically, the Pterasteridae (Fig. 6E) is the most diverse within the Velatida including 116 species in 8 genera [74]. Nearly all pterasterids occur in either cold or temperate water habitats, especially in the deep-sea or at high-latitudes in Arctic and Antarctic regions. One exception is the widely distributed *Euretaster*, which occurs in tropical, shallow-water settings throughout the Indo-Pacific.

### The Concentricycloidea

The Concentricycloidea, initially included the South Pacific *Xyloplax medusiformis* [7] and later came to include the tropical Atlantic *X. turneri* [8]. The original authors perceived the Concentricycloidea as morphologically distinct enough to warrant recognition at the class level.

Rowe et al [8] hypothesized that *Xyloplax* was “derived from asteroid asterozoans, possibly from a common ancestor of certain valvatids…” but clarified that “…the degree of developmental and morphological shift is such that it cannot be defined as a member of the class Asteroidea.” Work on spermatozoon morphology, spermiogenesis and microstructure [17], [287] were used to further argue the distinctiveness of Concentricycloidea as a separate class.

Following these initial reports, subsequent studies of *Xyloplax* classification emphasized phylogenetics, using cladistics to analyze synapomorphies, i.e., unique characters or molecular data that support a clade. Smith [288] placed concentricycloids within the Asteroidea, proposing shared synapomorphies between *Xyloplax* and the caymanostellid, *Caymanostella*. Pearse and Pearse [289] were the first to perform a phylogenetic analysis of *Xyloplax* along with other Echinodermata. Their results were equivocal, but they were unable to support submerging *Xyloplax* within the Asteroidea as proposed by Smith [288].

Janies and Mooi [290] and Janies [176] provided the first molecular/combined data analyses to include *Xyloplax*. Janies' tree supported *Xyloplax* as a derived branch, on the same branch as the asteriid *Rathbunaster*, within the Asteroidea using 18S and 28S rDNA sequences. Janies et al [173] later presented a molecular phylogeny, including data from seven loci (18S rRNA, 28S rRNA, histone H3 from the nucleus, 16S rRNA, 12S rRNA, cytochrome c oxidase subunit I, tRNA-Ala, tRNA-Leu, and tRNA-Pro of the mitochondrion), which placed *Xyloplax* as a sister taxon to a branch containing *Hymenaster* and *Pteraster*. Janies et al. [173] and Janies and McEdward [291], [292] argued that concentricycloids were progenetic velatid asteroids based on studies of larval asteroid morphology.

Mah [293] described a third species, *Xyloplax janetae* and presented a position intermediate between retaining *Xyloplax* as a separate class [7], [8] and inclusion within the Asteroidea [173], [288] by placing Concentricycloidea within the asteroid lineage, but as a sister-group to the Neoasteroidea, the group including all living asteroids. This placement is consistent with the hypothesis of an evolutionary bottleneck at the Permian-Triassic transition [168], which may have resulted in the extinction of *Xyloplax*'s closest sister taxa.

Mah [293] does not necessarily disagree with new phylogenetic data. Separation of the Velatida from other asteroid groups and its possible position as sister taxon to the other asteroid groups on the tree is a new one. However, members of the Velatida possess several autapomorphies, such as the absence of a clear marginal series, the absence of pedicellariae, and the lack of actinal plates, that set the group apart from other neoasteroids. These have historically been interpreted as highly derived [4], [5] but taken in the context of Janies et al., [173] and the phylogenetic trees presented by Mah and Foltz [69], [181] the Velatida display prominence as a distinct group within the Neoasteroidea, separate from the Forcipulatacea and the Valvatacea. Janies et al. [173] supported *Xylopla*x as the sister group to other living velatidans. If the Velatida were to be supported as the sister-group to the remaining Neoasteroidea then Mah's placement of *Xyloplax* (including the Velatida) would be consistent with the basal position of *Xyloplax* as presented by Janies et al. [173] but not necessarily as the sister group to the Neoasteroidea. However, identification of the definitive sister group to modern asteroids from fossil morphology [4], [5], [111] remains unresolved and in need of continuing efforts. Definitive sister-group rooting for asteroid phylogeny using molecular data is premature with many obstacles, including taxon sampling and identification of long-branches that have yet to be overcome [294].

### Extinct Groups

Most of the larger extant families of asteroids have been recognized in the fossil record, and although a few extinct families have been recognized, these are not large and do not differ greatly from those that do survive. Although fossil asteroids can be found all over the world, fossil deposits from the Mesozoic, especially the Cretaceous of Europe are among the most heavily studied and the best known. Accounts below are limited to extinct higher taxa with no surviving members.

Perhaps largely reflecting their modern occurrences and robust construction, most fossil taxa have been assigned to either Valvatida or Paxillosida. Included among extinct families is the Pycinasteridae is a small family, occuring primarily in the Mesozoic and early Cenozoic [94] that shows affinities with the Goniasteridae. The Stauranderasteridae has recently been reviewed by Villier et al. [295] and displays some morphological features that are reminiscent of the Oreasteridae. Paleobiology of stauranderasterids is poorly known, but at least some taxa have been collected from Jurassic tropical, shallow-water sediments [93]. The Mesozoic Sphaerasteridae was considered convergent with living *Podosphaeraster* by Blake [270] and were formally separated by Fujita and Rowe [251]. Relatively few recent accounts of fossil sphaerasterids [296], [297] are available.

Although the Goniasteridae is extant, a significant number of goniasterid genera occur only as fossil. A total of 102 living and extant goniasterid genera are recognized. Goniasterids can be broken down into three groups: 57 are known only from the extant, 8 are known from both living and fossil, and 37 are fossil-only genera. No other post-Paleozoic asteroids have such a significant number of taxa contributing to the overall diversity.

Among the non-valvatidan fossil groups within the Valvatacea is the Paleobenthopectininae [214], whose members were supported as the sister group to the extant Benthopectinidae within the Notomyotida as reconstructed by Blake [4]. Mah and Foltz [69] placed the benthopectinids as a lineage within the Paxillosida. Villier et al. [95] allied the Paleobenthopectininae as members of the Velatida with members showing affinities with the Myxasteridae. Blake et al. [169] described *Noriaster*, an early member of the Poraniidae from the Triassic of Northern Italy.

Within the Velatida is the monotypic Jurassic Tropidasteridae which Blake [298] supported as phylogenetically near velatidans, such as the Myxasteridae, Korethrasteridae and the Pterasteridae.

The Trichasteropsida is a member of the Forcipulatacea [4], [6] and occupies a basal position relative to other forcipulataceans, both owing to its phylogenetic position and its Triassic fossil occurrence, which places its two members, *Trichasteropsis* and *Berckhermeraster* among the earliest of post-Paleozoic fossil asteroids [6]. Gale [111] established the monotypic Terminasteridae within the Forcipulatida.

### Conclusions and Future Research

Asteroid biodiversity and systematics remains an active area of research that has brought additional depth to our understanding of echinoderm evolution and historical changes in the marine setting.

The use of molecular tools to infer asteroid phylogeny and classification is still comparatively new, nevertheless significant changes have already emerged and this trend can be expected to continue at all taxonomic levels. For example, classification within the Asteriidae had been problematic since Fisher's [101], [183] revision of the Forcipulata. The recent revision of the Forcipulatacea by Mah and Foltz [181] shows strongly supported lineages within the Asteriidae that are not immediately obvious from external morphology. Zulliger and Lessios [83] presented a molecular phylogeny of the species-rich *Astropecten*, including taxa collected from throughout its range. Their work identified multiple species complexes and recognized morphological and ecological convergence among taxa present throughout *Astropecten*'s global distribution.

Historically, interpretations of phylogeny have been based primarily on morphology, although early ontogeny has also played a significant role. Molecular phylogenetics circumvents the circularity of using morphology for interpretation of both phylogenetic history and functional phylogenetic changes. For example, taxa such as *Pseudarchaster* possess morphological adaptations that suggest living on unconsolidated sediment (e. g., presence of paxillae, well-developed fasciolar channels, etc.). However, emphasis on certain characters (e.g., suckered tube feet) has historically placed *Pseudarchaster* (and other pseudarchasterines) within the Valvatida precluding their inclusion within the Paxillosida, which has been historically defined by the presence of pointed tube feet. The molecular phylogeny of the Valvatacea by Mah and Foltz [69] supported *Pseudarchaster* as a member of the Paxillosida, running contrary to its traditional taxonomic position.

New collections of specimens from marine exploration continue to provide further data for our understanding of biodiversity in shallow-water and deep-sea settings. Additional sampling has not only added to our discovery of undescribed biodiversity [200], [229], [254], [257], [293] but has also provided us with new measures of zonation and abundance [299]. The availability of video has also brought an unprecedented wealth of ecological data from high resolution, *in situ* observations [100].

The fossil record is meager, but field and museum research continues to reveal important discoveries about the earlier history of asteroids, and can be expected to continue to do so.

In spite of the considerable progress, which has been summarized herein, several topics remain crucial for future research.

1. **Basal phylogenetic relationships.** In spite of comprehensive phylogenetic efforts, such as those of Mah and Foltz [69], [181] basal relationships among major lineages of asteroids remains a contentious subject. Support for early divergence of asteroid lineages has been elusive, pending discovery of more conserved genetic markers that will permit inference of basal relationships. Also important to understanding the early diversification of modern asteroids are fossils from the early Mesozoic/late Paleozoic that provide further evidence for early diversification of the crown-group.
2. **Problematic groups.** Xyloplax *and* Podosphaeraster. Current data from molecular phylogenies has not settled the phylogenetic questions regarding these enigmatic taxa and little is known regarding the biology and development of these highly unusual asteroids. These questions are, in part, tied to development of a well-supported phylogeny of the Asteroidea, which is concern #1 (above).
3. **Undiscovered Biodiversity.** A large potential exists for undiscovered asteroid taxa. This includes the potential for cryptic species that will likely be discovered in widely occurring deep-sea taxa. Museum collections of taxa from improved and increased expeditions, as well as living and fossil collections will also become important as unidentified material is processed.
